# The CHEK 2 GENE mutations and the risk of Gastric cancer

**DOI:** 10.1186/1897-4287-10-S1-A10

**Published:** 2012-01-12

**Authors:** Urszula Teodorczyk, Cezary Cybulski, Anna Jakubowska, Teresa Starzyńska, Małgorzata Ławniczak, Katarzyna Ferenc, Krzysztof Marlicz, Zbigniew Banaszkiewicz, Rafał Wiśniowski, Jan Lubiński

**Affiliations:** 1 International Hereditary Cancer Center, Department of Genetics and Pathology, Pomeranian Medical University, Szczecin, Poland; 2 Clinic of Gastroenterology, Pomeranian Medical University, Szczecin, Poland; 3 Clinic of General Surgery, University Hospital, Bydgoszcz, Poland; 4 Beskidian Center of Oncology, Bielsko Biała, Poland

## Background and aims

CHEK2 gene is located on chromosome 22q12.1. and encodes the human analogue of the yeast checkpoint kinases Cds1 and Rad 53. Activation of CHEK2 in response to DNA damage prevents the cell from entering into mitosis. Three founder alleles are present in Poland. Two of these result in a truncated CHEK2 protein IVS2+1G>A in exon 3 and 1100 del C in exon 10, the other, I157T is a missense substitution of an isoleucine for a threonine in exon 3. A single founder allele of the CHEK2 has been associated with predisposition to breast and prostate cancer in North America and Europe. CHK2 alterations are associated with an increased risk of thyroid, prostate, breast, colon and kidney cancer in Polish population. Recently, a large deletion of exons 9 and 10 of CHEK2 was identified in several unrelated patients with breast cancer of Czech or Slovak origin, the del 5395 also confers an increased risk of prostate cancer in Polish men. The CHEK2 is therefore a good candidate for a multisite cancer susceptibility gene. We reasoned, that CHEK2 alterations ought to be investigate in gastric cancer cases, too.

## Patients and methods

We have examined the frequency of the CHEK2 gene mutations in a series of randomized individuals including 749 consecutively collected stomach cancer , 166 patients with familial gastric cancer, and 5496 control patients. The 1100 del C, IVS2+1G→A, I 157 T and del 5395 mutations were identified by ASA-PCR, RFLP-PCR, multiplex-PCR.

## Results

The frequency of the I157T mutation in a group of consecutive gastric cancer patients was significantly elevated compared to the control population (OR=1.418, p=0.0348), herein in the group of patients diagnosed with disease less then 50 years of age (OR=1.825, p=0.0511). I157T was over-represented in the group of familial gastric cancer patients (OR=2.246, p=0.003 ) too, herein in patients diagnosed with disease less then 50 years of age (OR=3.171, p=0.0044) and in females (OR=2.973, p=0.0041).

The IVS2+1G>A was over-represented in a group of consecutive patients with gastric cancers ( OR=3.367, p=0.002), therein in patients diagnosed under 50 years of age (OR=4.524, p=0.0377) and over 50 years of age (OR=3.034, p=0.0183 ) additionally in males (OR=3.706, p=0.0041). A large deletion of exons 9 and 10 confers an increased risk of familial gastric cancer in patients diagnosed over 50 years of age (OR=5.922, p=0.0598), but this result is not quite significant.

## Conclusions

The CHEK2 I157T mutation may be a predisposing genetic factor associated with both, consecutive and familial gastric cancer risk. Occurrence of IVS2+1G→A alteration confers an increased risk of consecutive gastric cancer.

